# Iatrogenic Circumflex Artery Stenosis Following Mitral Valve Repair

**DOI:** 10.7759/cureus.1680

**Published:** 2017-09-12

**Authors:** Tatiana Busu, Fahad Alqahtani, Akram Kawsara, Mohamad Hijazi, Mohamad Alkhouli

**Affiliations:** 1 Heart and Vascular Institute, West Virginia University School of Medicine/Ruby Memorial Hospital; 2 Division of Cardiovascular Disease, West Virginia University School of Medicine/Ruby Memorial Hospital

**Keywords:** mitral valve repair, iatrogenic injury, left circumflex, optical coherence tomography

## Abstract

Injury of the left circumflex coronary artery is a potentially serious complication of mitral valve surgery due to the proximity of the vessel to the posterior segment of the mitral annulus. Suture-related distortion of the artery with partial or subtotal occlusion is the most commonly implicated mechanism. Herein, we present a case of symptomatic iatrogenic circumflex coronary artery stenosis following mitral valve annuloplasty for degenerative mitral valve regurgitation.

## Introduction

An iatrogenic coronary artery injury following cardiac valve surgery is a rare but under-recognized entity. The proximity of the left circumflex coronary artery to the mitral annulus makes this artery particularly susceptible to hemorrhagic and nonhemorrhagic injuries during mitral valve repair and replacement surgeries [1]. An unintentional capture of the artery with the suture material during surgery can lead to an iatrogenic coronary stenosis. The recognition of this complication can be difficult due to the variable manifestation and the lower threshold of subject patients with previously normal coronaries to further testing [2]. We present a case of an undiagnosed iatrogenic left circumflex coronary artery following mitral valve repair that manifested with recurrent effort angina. In this manuscript, we aim to raise awareness of this rare but potentially debilitating complication. 

## Case presentation

**Figure 1:**
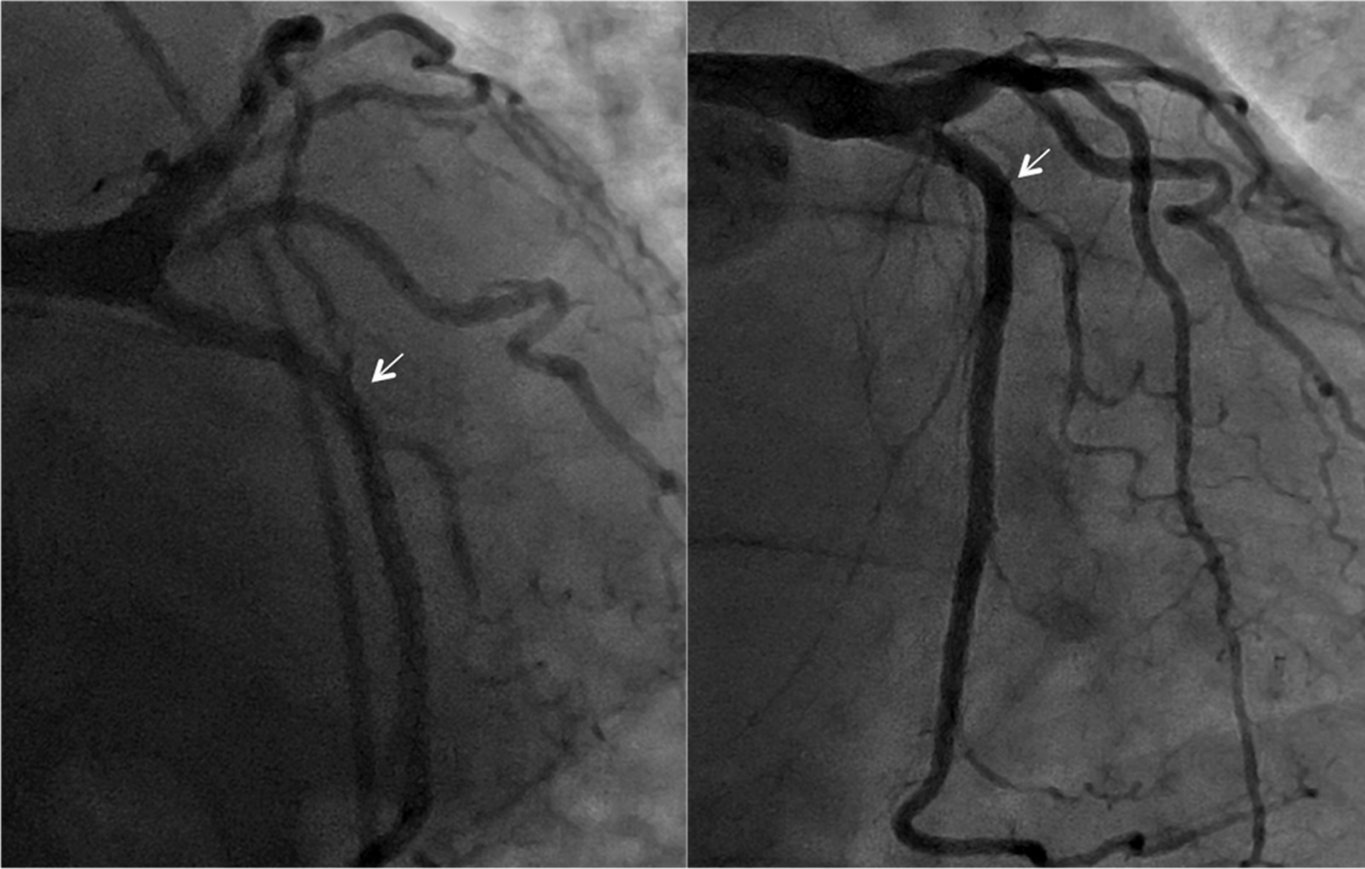
Preoperative coronary angiography showing no obstructive disease in the left circumflex coronary artery

A 67-year-old male presented with recurrent exertional chest pain. The patient has a history of atrial fibrillation and severe degenerative mitral regurgitation but no history of coronary artery disease. Two years prior, he underwent mitral valve repair with a quadrangular resection of the posterior leaflet and ring annuloplasty with a 34-mm Memo 3D annuloplasty ring (Sorin Group, Italy), a modified maze procedure, and an exclusion of the left atrial appendage with AtriCure (AtriCure, Mason, OH). His preoperative angiogram showed no significant coronary disease (Figure 1).

**Figure 2:**
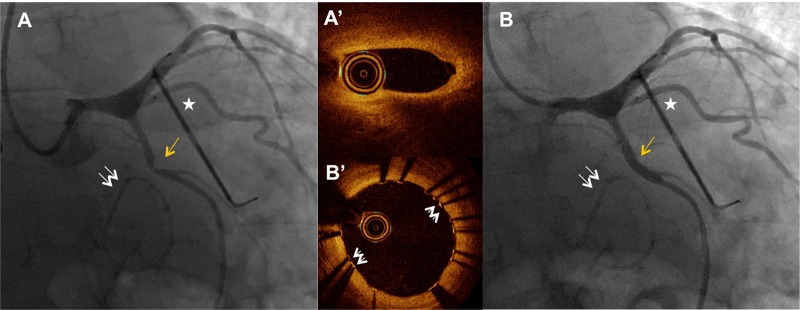
Iatrogenic circumflex stenosis adjacent to the mitral ring 2A - Postsurgery coronary angiography showing severe mid circumflex stenosis (yellow arrow). Double arrows indicate the mitral ring and the star indicates the AtriCure left atrial appendage occlusion device. 2A’ - Optical coherence tomography showing the site of iatrogenic stenosis. 2B - Post-balloon angioplasty and stenting angiography showing resolution of the stenosis. 2B’ - Optical coherence tomography after stenting showing a well-apposed stent and no residual stenosis. The white arrows annotate the stent struts.

Due to his recurrent chest pain, a repeat angiogram was done, which showed severe stenosis and distortion of the mid circumflex artery in close proximity to the suture line of the mitral ring (Figure 2A). The lesion was crossed with a 0.014" Runthrough wire with moderate difficulty (Terumo, Somerset, NJ). Optical coherence tomography (OCT) was then performed with a Dragonfly OPTIS imaging catheter (St. Jude Medical, Minnesota, United States) and showed no significant atherosclerosis in the circumflex artery but a severe iatrogenic stenosis of the mid segment of the artery (Figure 2A’). The lesion was dilated with a 2.5x12 mm Sprinter RX balloon (Medtronic, Minnesota, United States) and stented with a 3.5x15 mm Xience stent (Abbott Vascular, Santa Clara, CA) (Figure 2B). Post-stenting angiography and OCT imaging demonstrated complete resolution of the stenosis and a well-apposed stent (Figure 2B’).

Following the intervention, the patient’s chest pain has resolved and he remained free from chest pain at the four weeks follow-up.

## Discussion

Iatrogenic coronary artery stenosis is a rare complication of mitral valve surgery due to the close proximity of the circumflex artery to the mitral valve annulus [3-4]. Various underlying causes of injury have been proposed; laceration of the artery can lead to both hemorrhagic complications as well as a thrombotic occlusion due to an injury of the endothelium, both with a potentially acute onset of symptoms. A more insidious onset of symptoms usually occurs with suture fixation with either complete encircling with occlusion of the artery or distortion of vessel anatomy due to suture fixation, or due to a dynamic occlusion secondary to spasm and/or subintimal hematoma [5]. Among all these potential causes, a suture-related injury is considered the most common etiology of iatrogenic coronary injuries [4].

The underlying etiology can be often suggested with coronary angiography. However, intravascular imaging with intravascular ultrasound or OCT can bring further anatomical details and aid in the ascertainment of that etiology. In this case, OCT revealed the absence of atherosclerosis, intimal or subintimal injury, and vessel wall hematoma and confirmed that the likely etiology was a suture-related encircling of the vessel. In this case, percutaneous balloon angioplasty and stenting were feasible and resulted in immediate symptomatic relief.

## Conclusions

The close anatomical proximity of the circumflex coronary artery to the posterior mitral annulus presents a risk of an iatrogenic injury of the vessel during surgical mitral valve repair or replacement. Increased awareness of this potential complication can aid in early identification and effective treatment.
